# Cervical screening: ESGO-EFC position paper of the European Society of Gynaecologic Oncology (ESGO) and the European Federation of Colposcopy (EFC)

**DOI:** 10.1038/s41416-020-0920-9

**Published:** 2020-06-08

**Authors:** Maria Kyrgiou, Marc Arbyn, Christine Bergeron, F. Xavier Bosch, Joakim Dillner, Mark Jit, Jane Kim, Mario Poljak, Pekka Nieminen, Peter Sasieni, Vesna Kesic, Jack Cuzick, Murat Gultekin

**Affiliations:** 1 Department of Surgery and Cancer - Gut, Metabolism and Reproduction IRDB, Faculty of Medicine, Imperial College London, London, W12 0NN UK; 2 West London Gynaecological Cancer Centre, Queen Charlotte’s and Chelsea – Hammersmith Hospital, Imperial College Healthcare NHS Trust, London, W12 0HS UK; 3 Unit of Cancer Epidemiology, Belgian Cancer Centre, Sciensano, Brussels, Belgium; 4 Department of Pathology, Laboratoire Cerba, 95066 Cergy Pontoise, Cedex 9 France; 5 Cancer Epidemiology Research Program, Catalan Institute of Oncology (ICO), Barcelona, Spain; 6 Bellvitge Biomedical Research Institute (IDIBELL), Barcelona, Spain; 7 Universitat Oberta de Catalunya, Barcelona, Spain; 8 Department of Laboratory Medicine, Karolinska Institut, Stockholm, Sweden; 9 Department of Infectious Disease Epidemiology, Faculty of Epidemiology and Population Health, London School of Hygiene & Tropical Medicine, London, WC1E 7HT UK; 10 Modelling and Economics Unit, National Infections Service, Public Health England, London, NW9 5EQ UK; 11 School of Public Health, University of Hong Kong, Hong Kong, SAR China; 12 Harvard T.H. Chan School of Public Health, Center for Health Decision Science, 718 Huntington Avenue, Boston, MA 02115 USA; 13 Institute of Microbiology and Immunology, Faculty of Medicine, University of Ljubljana, Ljubljana, Slovenia; 14 University of Helsinki, Helsinki, Finland; 15 King’s Clinical Trials Unit, King’s University, London, UK; 16 Medical Faculty, University of Belgrade; Clinic of Obstetrics and Gynecology, Clinical center of Serbia, Beograd, Serbia; 17 Wolfson Institute of Preventive Medicine - Barts and The London, Queen’s Mary University, Charterhouse Square, EC1M 6BQ London, UK; 18 Division of Gynaecological Oncology, Department of Obstetrics and Gynaecology, Hacettepe University Faculty of Medicine, Ankara, Turkey

**Keywords:** Health policy, Education, Preventive medicine

## Abstract

This paper summarises the position of ESGO and EFC on cervical screening based on existing guidelines and opinions of a team of lead experts. HPV test is replacing cytology as this offers greater protection against cervical cancer and allows longer screening intervals. Only a dozen of HPV tests are considered as clinically validated for screening. The lower specificity of HPV test dictates the use of triage tests that can select women for colposcopy. Reflex cytology is currently the only well validated triage test; HPV genotyping and p16 immunostaining may be used in the future, although methylation assays and viral load also look promising. A summary of quality assurance benchmarks is provided, and the importance to audit the screening histories of women who developed cancer is noted as a key objective. HPV-based screening is more cost-effective than cytology or cotesting. HPV-based screening should continue in the post-vaccination era. Only a fraction of the female population is vaccinated, and this varies across countries. A major challenge will be to personalise screening frequency according to vaccination status. Still the most important factor for successful prevention by screening is high population coverage and organised screening. Screening with self-sampling to reach under-screened women is promising.

## Background

Cervical cancer is largely preventable through local treatment of screen-detected cervical preinvasive lesions (high-grade cervical intraepithelial neoplasia, HG CIN). Progression from HPV infection to cancer takes normally 15–20 years. This long natural history with a prolonged precancerous phase permits early detection and treatment through population screening. Despite screening, invasive cervical cancer remains the commonest female neoplasia in many sub-Saharan African countries and the fourth most common cancer in women globally. An estimate of 570,000 women developed and 311,000 died from cervical cancer in 2018. Of all cervical cancers, 84% of cases and 88% of deaths occur in lower resource countries (human development index <80) where access to preventive care is poor.^1^

Countries with organised screening programmes have reported significant reduction in the incidence and mortality from cervical cancer as a result of treatment of screen-detected lesions.^2^ Inevitably it also leads to the early detection of some cancers (that it fails to prevent), which also has a benefit in reducing mortality from cervical cancer. In the UK, incidence of cervical cancer dropped by 24% since the introduction of a national screening programme in 1988.^3^ Mortality has dropped from 8/100,000 in 1988 to 3/100,000 in 2017.^3^ In Finland, after introducing the population-based screening in the 1960s, the incidence and mortality have both dropped by 80%. Incidence is presently 4/100,000 and mortality 1/100,000 women years.^4^ The incidence and mortality trends across counties in Europe and beyond depend on a number of factors from HPV prevalence and ecology to the availability, quality and coverage of screening programmes.

Within the last decade we have seen major advances in cervical cancer prevention both within the proposed screening strategies with the introduction of HPV DNA test as well as the implementation of prophylactic HPV vaccination. This position paper jointly produced by the European Society of Gynecologic Oncology (ESGO) and the European Federation of Colposcopy (EFC) summarises major advances and how these are likely to influence future screening programmes. The statements presented in the paper present summaries of existing guidelines and the best available evidence. If these are not available, the opinion of a team of lead experts in cervical cancer screening and prevention is presented. The references to existing guidelines are included in the relevant sections. In order to produce this statement paper, one or two experts for every section presented in this position paper have been invited to perform a literature search and produce a summary chapter. Each of these chapters were subsequently summarised in this statement paper.

## Role of cytology after over 50 years of experience globally

Exfoliative cytology has been the mainstay for cervical screening. Traditionally, the cells were taken by a spatula or a brush, smeared on glassed slides and stained as first described by Papanicolaou in the ‘40 s. More recently, this has been replaced in some countries by liquid-based cytology (LBC) where the cells are collected with a plastic brush suspended in a fixative-solution. Two collection devices have been validated by Food and Drug Administration (FDA). LBC has a number of advantages. LBC allows a semi-automated production of slides and has the ability to remove debris, red cells, inflammatory cells and artefacts producing a uniform thin spread of epithelial cells that is easier to read by cytopathologists and cytotechnicians. LBC has been shown to reduce the rate of unsatisfactory smears from 9.1% to 1.6% in some settings,^5^ while the solution can be used for reflex testing for HPV test and other markers. A meta-analysis conducted as part of the European Guidelines for Quality Assurance in Cervical Cancer Screening concluded that although liquid-based and conventional cytology had similar sensitivity and specificity for the detection of CIN2 or worse (CIN2+) at all cytological cut-offs except at cut-off atypical squamou cells of uncertain significance (ASC-US) (where LBC tended to be less specific), LBC was found to improve quality and speed of interpretation allowing further molecular testing.^6^

The Bethesda terminology first introduced in 1988 is used for cytology reporting and was updated in 2001 and more recently in 2014. The majority of cervical abnormalities are in squamous cells. Atypical squamous cells of undetermined significance (ASC-US) and low-grade intraepithelial lesions (LSIL) represent the majority of abnormal smears with rates of around 3–5% and 1–2% respectively, while only 0.5–1% of smears are described as high-grade (HSIL). Glandular cells abnormalities are rare and represent 0.2% of all smears and less than 4% of abnormal smears.^7^

Cytology has also limitations with a 20–25% of false-negative results. Review of new cervical cancer cases revealed most cases (60%) are in women non-compliant with screening, while another 10% in woman managed inappropriately. However, one third of new cancer cases (30%) are women that attended screening and received a negative result due to incorrect sampling or interpretation.^8^ Furthermore, cytology has limitations in detecting glandular intraepithelial lesions located in endocervical glands.^9^ This is because many do not reach the superficial part of the gland and do not shed exfoliative cells. The incidence of glandular disease and adenocarcinomas has been progressively increasing in some countries with those comprising over 20–30% of cervical tumours. These cancers have poorer prognosis, partly reflecting delays in diagnosis and higher clinical stage.

## HPV test in primary cervical screening

Many countries are switching to high-risk HPV (hrHPV) testing for cervical cancer screening, at least in women above the age of 30 and major reorganisation of existing screening strategies is anticipated.

### Advantages and disadvantages

The use of hrHPV tests as the primary screening modality has several advantages. Several randomised controlled trials and a meta-analysis of randomised data reported Level A evidence that HPV tests have substantially higher sensitivity and negative predictive value in the detection of high-grade disease and when compared to cytology. HPV-based screening has a 60–70% better protection against invasive cervical cancer in women over the age of 30 when compared to cytology.^9,10^ The benefits are particularly evident in glandular disease. The higher sensitivity permits longer screening intervals, typically 5 years after a negative result, as opposed to the interval for cytology of 3–5 years or even less in some countries. hrHPV is an objective test with low inter- and intra-variability. The test can be run in central laboratories to ensure quality assurance and requires virtually no technical knowledge for reliable results. This alleviates the need for trained cytotechnicians that necessitate training and continuous revalidation for quality assurance that is not feasible in less affluent settings. hrHPV tests further reduces the number of unsatisfactory results at screening and permits self-sampling with comparable sensitivities to physician-collected samples, albeit a slightly lower specificity. Self-sampling may be particularly important for poor compliers and women in rural areas with limited access to health centres.^11^

The major disadvantage of hrHPV testing is its very age -dependent lower specificity when compared to cytology as the test can detect transient HPV infections without a true carcinogenic potential.^12,13^ The use of hrHPV primary screening in women under 30 years of age is not advised, because of the high prevalence of hrHPV infections in this age group. To improve specificity and minimise over-referral to colposcopy, triage tests are needed to identify infections more likely to be persistent and associated with the development of cancer. Whereas in the past hrHPV testing was expensive, prices of the former have dropped substantially and in some countries with tender procedures for purchasing HPV testing has become cheaper than cytology. Cost-effectiveness analyses have shown that primary hrHPV-based screening is more cost-effective than cytology-based screening as the higher cost of HPV testing is to some extent offset by its higher detection rate and consequent ability to safely screening at longer intervals between tests.^9,14,15^ HPV test may also have adverse psychological sequelae in women that test positive at screening; the type and severity of these will depend on cultural and religious factors and are country-specific.^16^

hrHPV DNA assays can be applied on vaginal self-samples, which offers opportunities to reach women who do not participate in the regular screening programmes. Meta-analyses have shown that clinically validated PCR-based assays are as accurate on self-compared to clinician-taken cervical samples.^17,18^

### HPV-test characteristics

With the exception of Hybrid Capture 2 and Cervista, most tests used for HPV detection are PCR-based. Most amplified HPV DNA, major exception is the APTIMA test which is RNA-based. Most tests detect a consensus of 13 high-risk types (HPV16, 18, 31, 33, 35, 39, 45, 51, 52, 56, 58, 59 and 68) associated with cervical cancer and some other cancers. Most of the validated tests have very similar overall characteristics in terms of clinical sensitivity and specificity.

### Which hrHPV test to use

Cervical cancer screening program should adopt a hrHPV test for use as screening tool only if it has been clinically validated by demonstrating reproducible, consistently high sensitivity for CIN2+ and CIN3+ lesions, and only minimal detection of clinically irrelevant, transient HPV infections. There is consensus in HPV community that HPV tests (neither commercial nor in-house tests) that have not been clinically validated should not be used in clinical practice. HPV testing should be performed only on samples processed and analysed in qualified laboratories, accredited by authorised accreditation bodies and in compliance with international standards.^19^ The laboratory involved in HPV-based screening should perform a minimum of 10,000 HPV tests per year.^20^

As of December 2019, at least 253 distinct commercial tests for detection of alpha HPVs and at least 425 variants of the original tests are available at the global market.^20–22^ Unfortunately, only a subset of commercial HPV tests has documented clinical performance for agreed indications for HPV testing in current clinical practice. For more than 60% of HPV tests on the global market, no single publication in peer-reviewed literature can be identified.^22^

As multitude of hrHPV tests are available, regularly updated evaluation of their suitability for primary screening is essential. A recent systematic review,^23^ listed the hrHPV DNA tests that were either validated through randomised trials showing a very low incidence of cervical cancer after a negative hrHPV DNA test^9,13^ or fulfilling international equivalence criteria based on cross-sectional data.^24^ The international equivalence criteria are based on non-inferior cross-sectional accuracy of a new HPV test versus one of the two benchmark comparator tests (GP5+/6+ PCR-EIA or HC2) that have been validated in clinical trials and detect the same molecular targets, i.e. DNA of hrHPV types.

As of December 2019, a dozen of HPV assays can be considered as validated or partially validated for primary HPV-based cervical cancer screening.

## Triage of HPV-positive women: how to eliminate low specificity of HPV screening

HPV-based screening is expected to increase the number of women that will test positive at screening due to lower specificity, especially among younger women, while efforts should be made to optimise triage strategies to prevent overload of colposcopy clinics and unnecessary interventions from passenger transient infections. Triage tests include cellular assays such as cytological findings, p16 with or without Ki67 immunostaining (only on clinician-taken samples), genotyping, viral load and methylation assays (on either self- or clinician-collected samples).

### Cytology

Cytology is the only triage test, for which there is high-grade evidence regarding its suitability to this role. Cytology has been implemented in many settings as a triage test in women testing positive for hrHPV. Reflex cytology of ASCUS or LSIL is the threshold for referral to colposcopy in the European Guidelines, while, if normal, women are retested for HPV in 12 months or for cytology in 6–12 months.^25^

Although not yet recommended in the European guidelines, two additional triage tests have been widely evaluated in this context:

### HPV genotyping

This is available for HPV16 and 18 in many assays. Performance varies across genotypes and these need to be considered separately. HPV16 is present in 50% of high-grade lesions^26^ with a PPV as a triage of about 15–20%. HPV33 typically has a similar or higher PPV than HPV16, but it is much less common. HPV31 also emerges as a type with a high PPV, is more common than HPV 33 and could therefore be used to select women who need immediate colposcopy.^27^ However, the role of HPV16 as the cause of precancer significantly decreases in older women compromising its use in triage.^28^ HPV18 genotype that is the second most common type in cancer after HPV16 does not perform well in triage. HPV18-associated lesions are common after over 5-year follow-up and most commonly in the endocervix associated with adenocarcinomas, dictating that different approaches might be considered for these lesions. Although this is currently an acceptable triage test in some countries like the US,^29^ recently published evidence from the English pilot in the UK suggest that 16/18 HPV genotyping in the presence of high-quality cytology and adherence to early recall, does not improve detection of CIN2 or worse.^30^

### p16^INK4a^ Immunostaining

Immunostaining for p16INK4a could be another useful option for the triage of HPV-positive women.^31,32^ Analysis from randomised evidence^33^ suggested that if only HPV and p16^INK4a^ immunostaining positive women were referred to colposcopy, referral rate would be similar to that of cytology screening with a 53% increase in detection of CIN2 or worse. More recently other studies have applied the p16^INK4a^/Ki-67 dual staining technology (CINtec plus, Ventana). Studies have shown that dual staining is more sensitive than cytology in detecting CIN2 or worse in HPV-positive women.^34,35^ It could allow a longer interval for HPV+ and p16/ki67− before referral to colposcopy. More studies are needed to clarify its role as a triage test after HPV primary screening.

Two further tests may prove of use in the triage of HPV-positive women:

### Methylation

There is ongoing research in the field. Only two tests have been fully evaluated. The first from a Dutch group^36^ uses hypermethylation of the promoter region of two human genes—the tumour suppressor genes FAM19A4 and/or hsa-mir124–2—and has been commercialised as the Qiasure test (by Qiagen). The second test by a UK group^37,38^ includes six genes and uses methylation of both the human genes EPB41L3, MAL1, the viral gene regions of HPV16L1/L2, HPV16E2BS, HPV18L2, HPV31L1 and HPV33L2.

### Viral load

Several papers have shown viral load to be an important quantifier of risk particularly of HPV16,^39–41^ but also other HPV types at different degree. Although available for many HPV tests (relative light units for HC2 and CT scores for PCR-based tests), further studies should validate its use to ensure values are appropriately adjusted for number of cells per sample.

### Future considerations

Although the possibility of combination test may improve accuracy of triage, these combinations may not apply or be possible for all tests and can be costly and time-consuming, and hierarchical modelling may allow multiple testing only for equivocal results. With self-sampling becoming a possibility in some screening settings, emphasis should lie on viral (genotyping, methylation) rather than cytological triage markers, and other molecular tests (e.g. p16 and mRNA) may also be proved to be useful.^42^

## Quality assurance in cervical screening

European Guidelines for Quality assurance in Cervical Cancer Screening established principles of organised, population-based screening.^43^ The second edition published in 2008^8^ defined the following necessary components of an organised screening program: defined target populations (women 25–65 years of age) and screening intervals (3–5 years if using Pap-smears and 5–7 (10) using hrHPV-test), use of a population-based registry and appropriate recruitment measures, like personal invitations and place and date for the taking of the screening test, definition of the screening test, adequate facilities to perform this test, defined management algorithms and monitoring and evaluation of the process and impact of screening. A supplement of 2nd edition published in 2015 recommends the integration of new technologies such as HPV-based screening and vaccination in the programs for cervical cancer control.^19^

The success of any screening in general, regardless of the methodology applied, is directly related to quality control of the program, and above all high population coverage. The principles of quality control and key performance indicators should be known by all involved stakeholders and health professionals.

The recommendations shown in Table 1 describe a short version of the European Union Guidelines for Quality Assurance in Cervical Screening, with a few updates because of new evidence and specifications on quality indicators that follow the guidelines by ESGO/EFC experts (13,34).

**Table 1:** Summary of European Union Guidelines for Quality Assurance in Cervical Screening, completed with ESGO/EFC expert opinion.

1	Screening needs to identify the target population that is to be screened. This is the basis of population-based programs and is usually done by obtaining a list of individuals from a population registry belonging to the target age range. EU guidelines accept starting screening in the range 20–29 years but do not recommend screening before 25 years. Screening of cohorts vaccinated against HPV can start later but currently no specific European-wide guidelines exist for vaccinated cohorts.
2	The target population that is due for screening should have a personalised invitation to screening. This is the basis of organised screening programs. In addition to the lists with the target population, lists with actually performed screening tests (from whom and when) should be obtained from the screening laboratories. In order to be able to provide a time, date and place for a personalised appointment, an organisation that can take the samples is also needed. Main Quality Indicator: Invitation coverage—number of women in the target population due for screening that receive a personalised invitation with specified time and place/all women in the target population due for screening
3	Women who do not attend their screening appointment should be sent a new personalised invitation next year. Main Quality Indicator: Renewed invitation coverage—number of non-attending women who receive a new annual invitation/All non-attending women.
4	Women who have not attended after repeated invitations could be sent an HPV self-sampling kit. Main Quality Indicators: Test coverage—number of women in the target population that are recommended to be screened that are actually screened in the recommended interval/all women in the target population that are recommended to be screened. Test coverage can be calculated for any given length of time. When monitoring the effect of repeat invitations and sending of self-sampling kits calculating test coverage at increased lengths such as 5-year or 10-year test coverage is useful.
5	Organised screening with HPV testing is recommended until the age of 65 years. Women who have had a negative screening test at age 65 can exit the program, whereas non-attenders still could receive invitations beyond the age of 65. There is no current agreement on the age of initiation of screening.
7	Double screening with both HPV and cytology is recommended against. Primary HPV testing outside of an organised programme is also recommended against.
8	Sending of self-sampling kits to the entire population (i.e. not only to non-attenders) is currently not recommended.
9	It is recommended to prolong the screening interval for HPV-negative women, to ensure that the annual proportion of the population that require gynaecological investigation is kept reasonably low (to save resources and avoid possible side effects). The interval can be prolonged.
10	Women who are HPV-positive in primary screening should be triaged with cytology. Women with abnormal cytology should be referred for gynaecological investigation. Direct referral to colposcopy of all HPV-positive women is recommended against. Main Quality Indicator: Proportion of women HPV-positive in primary screening who are cytology-positive and are referred/all women who are HPV-positive in primary screening. HPV-positive women with negative reflex cytology should have a new triage test some time later, which could be cytology or a hrHPV test. Other options can be considered.
11	In age groups where primary cytology screening is used (below 30 years), women with high-grade cytology or worse should be directly referred. Women with equivocal or mildly abnormal may have a reflex HPV-test and be referred to colposcopy if HPV-positive. Screening programs should monitor the results of the program and allow for incremental optimisations in the program: Repeat testing of women with inconclusive screening (e.g. HPV + /cytology−), referral policies and compliance to referral, results of triage tests, colposcopies, biopsies and treatment of precancers. The efficiency of the program should be continuously monitored to ensure optimal use of resources that results in a maximal protection against cervical cancer. Key factors to monitor are the proportion of screen-positive women (the prevalence of HPV infections), the number and cost of invitations, sampling, testing and repeat testing, colposcopies and CIN treatments, in the context of the observed reduction in the incidence of cervical cancers.
12	All laboratories performing cytology or HPV testing should be accredited and take part in an official quality assurance program. The screening program should audit cancer cases.
13	When purchasing HPV tests for primary screening, programs should only ask for HPV tests that have been clinically validated.

At this stage it is unclear what should be the recommended age at first screen, the importance of the specifics of the local epidemiology (HPV prevalence by age) or even the optimal frequency of screening events. Moreover, although theoretically we anticipate that the screening requirements of the vaccinated cohorts will be significantly reduced, we have limited empirical data to consolidate formal ESGO recommendations. More documented guidance should be available as vaccinated cohorts arrive at a range of first screening age (i.e. 25–35) in the coming few years.

## Cost-effectiveness of cytology and HPV DNA for primary cervical cancer screening

Cost-effectiveness analyses can be used to integrate information about screening algorithms from trials and cross-sectional studies to project their long-term outcomes. Such analyses can show the trade-offs between greater reductions in cervical cancer cases and deaths on the one hand, and on the other hand, more positive tests, colposcopies and treatments, which may diminish women’s quality of life and increase costs.

In high-income countries, most economic evaluations have found using HPV test as the primary screen (whether alone or as a cotest with cytology) was more cost-effective than cytology alone.^44^ Most studies also show that HPV as the sole primary screen is more cost-effective than using it for cotesting with cytology.^45,46^ A key driver of cost-effectiveness is the fact that HPV DNA testing allows a longer interval between screens without increasing the risk of cancer.

However, results differ across settings and studies because of differing assumptions around key model inputs, such as:

- HPV prevalence: Lower population HPV prevalence (such as in vaccinated populations or older women) improves the cost-effectiveness of HPV testing when compared to cytology.^47,48^
- Costs: High-volume centralised laboratories can save costs.^49^
- HPV-test characteristics: HPV testing kit sensitivity and specificity and testing methodology (e.g. self-collected vs. provider-collected specimens) may influence cost-effectiveness and likely uptake.
- Quality of life: The assumed impact of positive HPV tests, colposcopies and treatment for neoplasia on quality of life can influence cost-effectiveness.^50^

Based on economic and other evidence, several countries have introduced or are in the process of introducing primary HPV testing into cervical screening algorithms (Table 2).

**Table 2:** Selected countries that have introduced primary HPV testing into cervical screening algorithms following economic and other evidence.

Country	Evidence review body	Status	Old algorithm	New algorithm	Economic evidence	Projected effect of switch
Australia	National Cervical Screening Programme	Introduced 2017	2-yearly cytology for 20–69-year-old women	5-yearly primary HPV testing with partial genotyping for 25–69-year-old women	^48^	Reduces cervical cancer incidence, mortality and costs by 31%, 36% and 19% in unvaccinated cohorts and 24%, 29% and 26% in vaccinated cohorts.
Netherlands	Dutch Health Council	Introduced 2017	3-yearly primary cytology for 30–60-year-old women	5-yearly primary HPV testing for 30–60-year-old women.	^62^	Prevents an additional 75 cervical cancer cases and 18 deaths a year without increasing costs.
UK	National Screening Committee	Pilot commenced in 2013, roll out by 2019	3-yearly cytology with HPV triage for 25–64-year-old women	3-yearly primary HPV testing with reflex cytology triage for 25–64-year-old women, possibly with partial genotyping. This may be extended to every 5-years in the future.	^63,64^	Reduces costs, cervical cancers and life years lost, but increases the number of colposcopies unless the interval between screens is lengthened.
Turkey	Ministry of Health, Turkey	Introduced in 2014	5-yearly cytology for 35–69-year-old women (low coverage)	5-yearly primary HPV testing for 30–65-year-old women	^65^ and M Gultekin, personal communication	
Italy	Ministry of Heath, ONS (Osservatorio Nazionale screening) Gisci	Pilot started in 2013, roll out by 2018 on a regional basis	3-yearly cytology for 25–65-year-old women	5-yearly HPV testing for 30–65-year-old women	^66–68^	

In conclusion, most studies suggest that HPV testing is more cost-effective than either cytology or cotesting as the primary screen. Cost-effectiveness analyses can help optimise primary HPV testing algorithms in their choice of triage test, interval between screens and age of screening in order to maximise the ratio of benefits to harms of screening within a particular setting.

## Screening in vaccinated populations

Cervical screening should continue in the era of HPV vaccination for the following reasons:

- The vaccine is purely prophylactic so that women already infected with an oncogenic HPV are not protected by the vaccine for that type. Since very few women (anywhere in the world) born before 1992 were vaccinated before they became sexually active, there will be a need to screen cohorts not offered vaccination for the foreseeable future (most likely until about 2060).
- Not all women receive the vaccine. Although unvaccinated individuals within vaccinated cohort may benefit from herd immunity, particularly when the coverage is high, it is clear that unvaccinated women will not be fully protected against HPV infection and cervical cancer for some years.^51^
- The current vaccines do not protect against all HPV types that cause cervical cancer. The two first generation vaccines offered excellent protection against HPV16/18 with some cross-protection against other among subjects who did not carry these HPV types.^52^ The new nonavalent vaccine (Gardasil9) with the additional five types (31/33/45/52/58) should prevent about 90% of cervical cancer in vaccinated women, but there is still a question on how to screen vaccinated cohorts
- Although there is no evidence of waning efficacy^53,54^ the vaccines are relatively new and there is no data on whether a vaccine given at age 12 will still protect women from infection 15–25 years later.

Corresponding to these reasons, there is a need to consider screening in four groups: cohorts not offered vaccination; unvaccinated women within vaccinated cohorts; vaccinated women and vaccinated cohorts in whom it is not known who has and has not been vaccinated.^55–57^ Additionally, continued monitoring of a cohort of women who were vaccinated early on is important so as to be able to adapt screening programmes should vaccine efficacy be shown to wane.

### Performance of HPV tests in vaccinated women

HPV vaccination provides an additional impetus for the use of HPV test in primary screening. If HPV type 16 and 18 infections, lesions and cancers are removed from the population, this will affect the test performance of both cervical cytology and HPV testing.^58^ HPV 16 and 18 account for about 70% of cancers but for just 30% of high-risk HPV infections in screened women.^59^ HPV16/18 prevalence in CIN2+ is in between these figures. Thus, the positive predictive value of both high-risk positivity and low-grade cytological abnormalities is greatly reduced in vaccinated women.^60^

Vaccination will reduce the prevalence of precancer in the screening population. That will mean that the numbers needed to screen to find one precancer will increase. Since cytology is subjective, this will cause additional challenges for cytoscreeners.^56^ In a cohort some of whom have been vaccinated, the specificity of HPV testing (and typing) at identifying those who have not been protected against HPV16/18 and are at greatest risk (because they have an HPV16/18 infection) is such that virtually everyone recommends HPV testing over cytology for such women.^61^

#### Cervical screening in birth-cohorts not offered vaccination

Screening will not require adjustment for women too old to have been offered HPV vaccination are unlikely to benefit from vaccination of younger women because birth-cohorts far apart in age are only weakly linked through sexual networks, so these women benefit little from herd protection, and because the majority of those who would be at risk of cervical cancer will have been infected by HPV before vaccination became widespread.

#### Cervical screening in unvaccinated women within vaccinated cohorts

Although herd protection is likely to reduce the risk of HPV infection and cervical cancer in unvaccinated women living in a vaccinated population, the magnitude and timing of such protection is still unclear. Women known not to have been vaccinated may be screened as before vaccination until there is greater evidence accumulates.

#### Women known to have been vaccinated

Since the lifetime risk of cervical cancer in women vaccinated against HPV is substantially lower than in unvaccinated cohorts, one could consider less frequent screening. Additionally, HPV 16 is the most carcinogenic HPV type, it makes sense to extend the screening interval and/or start screening at older age.^56^

#### Women in a partially vaccinated cohort

By lack of linkage between HPV vaccination and screening data basses it is impossible to differentially invite women depending on their individual vaccination status. As one would anticipate, the screening recommendations for partially vaccinated populations would be intermediary between those for unvaccinated and those for vaccinated populations.

In summary, screening will have to continue for decades to account for non-vaccinated/partially vaccinated cohorts. In HPV vaccine times HPV screening will replace cytology as primary screening test. Before several cohorts are intensively vaccinated, discrimination of screening protocols as function of the vaccination status of the individuals seems to be impractical in most settings. Protocols for HPV-based screening tend to target women over 30 and be offered every 5–7 years, a critical component of the sustainability of the programs under intensive research. Further ongoing research explores how impact of the vaccines can be accelerated with vaccination of adult women and high-risk groups, while reduction of cost and ensuring that there is no shortage of vaccines may further advance efforts for cervical cancer elimination in the future. Prophylactic vaccines do not have a role in women with existing HPV infections and/or preinvasive disease. A number of therapeutic vaccines in women with existing cervical disease are currently under investigation.

## Conclusions

In the era of rapid changes with HPV-based screening and prophylactic vaccination, screening strategies are undergoing major restructure. This document summarises the current position of ESGO and EFC in relation to cervical screening. More specifically, we discuss the advantages of HPV-based screening, the challenges in the triage of HPV-positive women and cost-effectiveness of HPV-based programmes. Quality assurance benchmarks are essential. HPV-based screening is likely to undergo further changes in vaccinated cohorts with prolonged intervals between tests. Education of women on the importance of screening should continue.

## Data Availability

Data sharing is not applicable to this article as no datasets were generated or analysed during the current study.
